# Halloysite-Nanotube-Mediated High-Flux γ-Al_2_O_3_ Ultrafiltration Membranes for Semiconductor Wastewater Treatment

**DOI:** 10.3390/membranes15050130

**Published:** 2025-04-27

**Authors:** Shining Geng, Dazhi Chen, Zhenghua Guo, Qian Li, Manyu Wen, Jiahui Wang, Kaidi Guo, Jing Wang, Yu Wang, Liang Yu, Xinglong Li, Xiaohu Li

**Affiliations:** 1 Beijing Key Laboratory of Photoelectronic/Electrophotonic Conversion Materials, Key Laboratory of Cluster Science, Ministry of Education, Advanced Technology Research Institute (Jinan), Beijing Institute of Technology Chongqing Innovation Center, Advanced Research Institute of Multidisciplinary Science, School of Chemistry and Chemical Engineering, Beijing Institute of Technology, Beijing 100081, China; 2 Beijing Institute of Technology, Zhengzhou Academy of Intelligent Technology, Zhengzhou 450000, China; 3 Chongqing Advanced Materials Institute (CAMI), Chongqing 408000, China; 4 Beijing Institute of Technology, Zhuhai 519088, China; 5 Guangdong Guoyu Equipment Co., Ltd., Foshan 528222, China; 6 School of Materials Science & Engineering, Beihang University, Beijing 102206, China

**Keywords:** chemical mechanical polishing wastewater, ceramic membrane, halloysite nanotubes, high flux, ultrafiltration

## Abstract

The wastewater from Chemical Mechanical Polishing (CMP) generated in the semiconductor industry contains a significant concentration of suspended particles and necessitates rigorous treatment to meet environmental standards. Ceramic ultrafiltration membranes offer significant advantages in treating such high-solid wastewater, including a high separation efficiency, environmental friendliness, and straightforward cleaning and maintenance. However, the preparation of high-precision ceramic ultrafiltration membranes with a smaller pore size (usually <20 nm) is very complicated, requiring the repeated construction of transition layers, which not only increases the time and economic costs of manufacturing but also leads to an elevated transport resistance. In this work, halloysite nanotubes (HNTs), characterized by their high aspect ratio and lumen structure, were utilized to create a high-porosity transition layer using a spray-coating technique, onto which a γ-Al_2_O_3_ ultrafiltration selective layer was subsequently coated. Compared to the conventional α-Al_2_O_3_ transition multilayers, the HNTs-derived transition layer not only had an improved porosity but also had a reduced pore size. As such, this strategy tended to simplify the preparation process for the ceramic membranes while reducing the transport resistance. The resulting high-flux γ-Al_2_O_3_ ultrafiltration membranes were used for the high-efficiency treatment of CMP wastewater, and the fouling behaviors were investigated. As expected, the HNTs-mediated γ-Al_2_O_3_ ultrafiltration membranes exhibited excellent water flux (126 LMH) and high rejection (99.4%) of inorganic particles in different solvent systems. In addition, such membranes demonstrated good operation stability and regeneration performance, showing promise for their application in the high-efficiency treatment of CMP wastewater in the semiconductor industry.

## 1. Introduction

The semiconductor industry relies heavily on Chemical Mechanical Polishing (CMP) to achieve the ultra-smooth surfaces required for device fabrication [1,2,3]. This process generates substantial amounts of wastewater containing high concentrations of suspended nanoparticles, abrasive materials, metal ions, and various organic additives [4,5]. The complex composition of CMP wastewater presents significant challenges for treatment, as the conventional methods often struggle to effectively remove ultra-fine particles while maintaining operational efficiency [6]. To comply with stringent environmental regulations, there is an urgent need for advanced separation technologies capable of efficiently treating CMP wastewater in a sustainable and cost-effective manner.

Ceramic ultrafiltration (UF) membranes have garnered attention as a promising solution for high-solid wastewater treatment due to their excellent chemical resistance, mechanical strength, thermal stability, ease of cleaning, and prolonged service life [7,8,9,10,11,12,13,14,15,16,17]. Compared to polymeric membranes, ceramic membranes exhibit a superior performance in harsh industrial environments, making them ideal candidates for CMP wastewater treatment. However, fabricating high-precision ceramic UF membranes with pore sizes below 20 nm remains a significant challenge [18,19]. The conventional approaches involve the sequential deposition of multiple layers [18,20,21,22,23,24], including a support layer made of α-Al_2_O_3_, followed by one or more transition layers that gradually reduce the pore size and finally a selective ultrafiltration layer. This multi-step process not only increases the complexity of fabrication and the production time but also introduces additional transport resistance [18,19,20,22,23,25]. Moreover, the repeated high-temperature sintering required to consolidate these layers often results in defects and inconsistencies in the pore size distribution, leading to high production costs and difficulties scaling up the fabrication process.

To address these persistent challenges, recent studies have explored using templating agents (e.g., carbon nanotubes, polymers) to engineer hierarchical pore structures [26,27,28]. Yet most methods still require complex post-treatment steps (e.g., sacrificial template removal), undermining the scalability of the process [19,29,30]. These challenges underscore the urgent need for innovative materials and manufacturing protocols that can decouple porosity enhancements from pore size reductions—a prerequisite for next-generation high-flux ceramic membranes.

Halloysite nanotubes (HNTs), naturally occurring aluminosilicate clays with unique lumen structures (of a 15–100 nm inner diameter) and high aspect ratios (>20) [31,32,33,34,35], present an intriguing solution to this conundrum. Unlike isotropic particles, the anisotropic geometry of HNTs enables their self-assembly into vertically aligned arrays during spray coating, creating interconnected macropores (~300 nm) while maintaining a narrow pore size distribution through nanotube stacking. Integrating HNTs into the membrane architecture can rapidly refine the pores and improve the overall porosity without the need for multiple deposition cycles. This approach simplifies the fabrication process and minimizes the transport resistance, addressing two critical limitations of conventional ceramic membranes: the trade-off between pore size control and overall permeability and the complexity of the multi-step fabrication procedures.

**Scheme 1:**
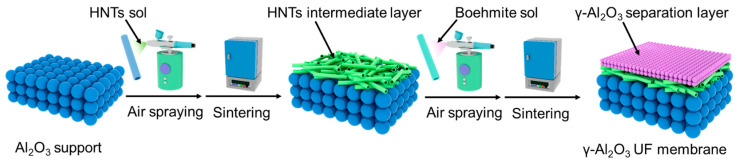
Illustration of the fabrication of HNTs-mediated γ-Al_2_O_3_ UF membranes.

In this study, we propose a novel, scalable strategy for industrial-scale production: the construction of a transition layer mediated by halloysite nanotubes (HNTs), followed by dip-coating with a γ-Al_2_O_3_ selective layer for semiconductor wastewater treatment (Scheme 1). Unlike the conventional methods that require multiple coating–sintering cycles (typically over four), our approach achieves an equivalent performance with a single coating–sintering step. This significantly simplifies the fabrication process, reduces the number of high-temperature sintering steps, and lowers both the overall complexity and cost while preserving the integrity of the membrane’s microstructure. The spray-coating process allows for the uniform and controlled deposition of the HNTs layer, resulting in a transition layer with a high-porosity and finely tunable pore structure. Furthermore, the proposed method takes advantage of three synergistic effects: (i) the lumen structure of the HNTs provides nanochannels to reduce the fluidic resistance, (ii) the hydroxyl-rich surface of the HNTs promotes the heterogeneous nucleation of the γ-Al_2_O_3_ nanoparticles, and (iii) the anisotropic packing of the HNTs creates size exclusion pores (<20 nm) at the inter-tubular junctions. To validate this concept, we systematically investigated the structure–property relationships of the HNTs-mediated γ-Al_2_O_3_ ultrafiltration membranes, elucidating their anti-fouling mechanisms and extended operation cycles under CMP wastewater treatment conditions. This work not only provides a scalable, energy-efficient pathway for ceramic membrane fabrication in the semiconductor industry but also establishes fundamental guidelines for the design of nanostructured transition layers in advanced separation systems.

## 2. Materials and Methods

### 2.1. Materials

The tubular Al_2_O_3_ support (average pore size, 1.5 μm; porosity, 45%) was kindly provided by Chongqing Advanced Materials Institute (CAMI, Chongqing, China), The pore size distribution of the membrane support is presented in Figure S1. The Al_2_O_3_ powders (200 nm, 1 μm) were supplied by SUMITOMO CHEMICAL Co., Ltd., (Tokyo, Japan). The HNTs were purchased from Yuan Xin Nano Technology Co., Ltd., (Guangzhou China). Ethanol (EtOH), silicon dioxide (SiO_2_), zirconium dioxide (ZrO_2_), Poly (Vinyl Alcohol) (PVA, M_w_ = 9000–10,000), and glycerol were purchased from Shanghai Titan Technology Co., Ltd., (Shanghai, China). Deionized (DI) water was used in all of the experiments. N_2_ (>99.999%) was purchased from Air Liquide and was used as delivered. All reagents and solvents were used without further purification unless otherwise specified.

### 2.2. Preparation of the High-Flux γ-Al_2_O_3_ Ceramic UF Membranes

#### 2.2.1. Preparation of the HNTs Intermediate Layer

The HNTs intermediate layer was prepared via air-spraying. Initially, the ceramic support was calcined at 800 °C for 0.5 h and subsequently boiled in DI water for 1 h to remove any possible contaminants. The support was then maintained at 100 °C and dried prior to further processing. A 10 wt% HNTs dispersion was prepared by dispersing the HNTs into a pre-prepared SiO_2_-ZrO_2_ sol [36]. The HNTs dispersion was uniformly sprayed onto the tubular support using a home-made air-spraying device with varying spraying durations and a spraying flow rate of 5 mL min^−1^. The coated support was dried at 110 °C for 20 min and then sintered in a muffle furnace at 600 °C for 20 min.

Membranes with multiple conventional α-Al_2_O_3_ intermediate layers were also prepared for comparison. Specifically, α-Al_2_O_3_ particles (5 wt% concentration) with different average diameters (1 μm, 0.2 μm) were dispersed into the pre-prepared SiO_2_-ZrO_2_ sols. The ceramic support was coated with the α-Al_2_O_3_ particles via the conventional wiping technique. The coated support was then dried at 110 °C for 20 min and subsequently sintered in a muffle furnace at 600 °C for 20 min. The coating–sintering cycles were repeated several times to guarantee the formation of high-quality intermediate layers.

#### 2.2.2. Preparation of the γ-Al_2_O_3_ UF Layer

To prevent the penetration of the boehmite sol, the surface of the pretreated support was first uniformly coated with a 10 wt% polyvinyl alcohol (PVA) solution and dried for 10 min. A certain amount of PVA was added to deionized water, heated at 95 °C for 2 h to melt the polymer, and then allowed to cool to room temperature, resulting in a 10% PVA aqueous solution. Subsequently, the support was coated with a 3 wt% boehmite sol for 30 s using the typical dip-coating method. After coating, the support was dried in a muffle furnace at 110 °C for 3 h, then sintered at 600 °C for 3 h, and finally cooled down to room temperature at a heating/cooling rate of 5 °C min^−1^. These steps were repeated several times. Detailed information on all of the ceramic membranes prepared are provided in Table 1.

### 2.3. Characterization of the Materials and Membranes

The morphologies of the Al_2_O_3_ powder and the HNTs were examined using scanning electron microscopy and transmission electron microscopy (TEM, JEOL, Tokyo, Japan), with the samples ultrasonically dispersed in ethanol prior to the analysis. Membrane samples were prepared by freeze-fracturing them after their immersion in liquid nitrogen, followed by platinum sputter-coating. A powder X-ray diffraction (PXRD) analysis was conducted using a Rigaku MiniFlex 600 diffractometer with Cu Kα radiation (λ = 0.154056 nm). The PXRD patterns were recorded over a 2θ range of 3–70° with a step size of 0.02° and a scan rate of 10° min^−1^. The N₂ adsorption isotherms were measured at 77 K using a Kubo-X1000 instrument, and the pore size distributions were determined through non-localized density functional theory (NLDFT). The thermal stabilities of the nanofillers and membranes were evaluated via a thermogravimetric analysis (PerkinElmer TG/DSC) at a heating rate of 10 °C min^−1^.

The open porosity of the ceramic membrane was evaluated using the Archimedean method. Initially, the membrane’s surface was cleaned, it was then dried in an oven at 110 °C for 12 h, and the mass of the dried membrane was recorded as *m*_1_. Subsequently, the dried membrane was immersed in deionized water and subjected to a vacuum for 30 min to ensure complete saturation. After saturation, the excess water on its surface was wiped off before its mass was recorded as *m*_2_. The porosity of the ceramic membrane was then calculated using Equation (1).

(1) $$\varepsilon =\frac{\left(m_{2}-m_{1}\right)}{\rho \pi h(r_{1}^{2}-r_{2}^{2})}\times 100\%$$

where *ε* denotes the porosity of the membrane (%), *m*_1_ and *m*_2_ denote the mass of the dry and wet membranes (kg), respectively, *r*_1_ and *r*_2_ denote the outer and inner diameters of the ceramic membranes (m), respectively, *h* denotes the length of the ceramic membranes (m), and *ρ* denotes the density of water, which is taken to be 1000 kg m^−3^.

For the prepared microfiltration membranes, the bubble point method was employed to measure their pore size and pore size distribution [37,38]. Initially, the samples were immersed in a wetting liquid to ensure that all of the pore channels were filled. Subsequently, the samples were removed and placed in the testing apparatus, where gas was gradually introduced. As the applied pressure increased, the gas progressively displaced the liquid from the pores, thereby opening the pore channels. According to the Laplace equation, the pore size could be determined using Equation (2).

(2) $$d=\frac{4\sigma \cos\theta }{\Delta p}$$

where *d* denotes the membrane pore size (m); *σ* denotes the surface tension of the wetting liquid (N m^−1^); *θ* denotes the contact angle (°); and Δ*p* denotes the transmembrane pressure difference (Pa).

The pore size distribution of the porous membranes was estimated using hexane or water as a condensable vapor based on the nanopermporometry method [39,40]. The principle of nanopermporometry is based on the capillary condensation of a vapor in porous membranes and its ability to block the permeation of a non-condensable gas (N_2_). This technique allows pores that are less than 50 nm in size to be measured. In the case of a capillary with a small pore size, vapor condenses at a vapor pressure (*p*) that is lower than the saturated vapor pressure (*p_s_*). The Kelvin diameter (*d_k_*), which increases with an increase in the vapor pressure of the condensable gas in the feed, was used to calculate the pore size.

(3) $$d_{k}=-\frac{4\sigma \nu \cos\theta }{RT\ln\left(p/p_{s}\right)}$$

where *σ* denotes the surface tension (N m^−1^), *ν* denotes the molar volume (m^3^/mol), *θ* denotes the contact angle (°), *R* denotes the universal gas constant, and *T* denotes the temperature (K).

### 2.4. The CMP Waste Liquid Filtration Test

The permeate fluxes of pure water, pure ethanol, and pure isopropanol at 25 °C were evaluated using a laboratory-made cross-flow membrane permeation performance test device.

(4) $$J=\frac{V}{A\;t}$$

where *J* (L m^−2^ h^−1^) is the pure liquid’s permeation flux; *V* (L) is the volume of the permeate, *A* (m^2^) is the effective filtration area of the membrane, and *t* (h) is the filtration time.

A custom-designed device was employed to evaluate the filtration performance of the membranes for semiconductor wastewater at 25 °C. A SiO_2_ solution with a SiO_2_ particle size of 50~100 nm was used to simulate the CMP waste liquids, and the concentration in the feed and the permeate was determined using a YC9200-4 full-parameter water quality rapid tester (Analytical Technology, Shenzhen, China). The retention rate of particulate matter was calculated according to Equation (5).

(5) $$R=\frac{C_{f}-C_{p}}{C_{f}}\times 100\%$$

where *R* is the retention rate; *C_f_* denotes the concentration of the feed solution (mg L^−1^); and *C_p_* denotes the concentration of the permeate (mg L^−1^).

The anti-fouling performance of the membrane was assessed through cyclic stability tests. In the first cycle, the initial permeate flux of pure liquid was measured before the membrane was used to filter the simulated semiconductor wastewater. The filtration was conducted under the following conditions: an applied pressure of 3 bar, a cross-flow rate of 20 L h^−1^, a temperature of 25 °C, and a filtration duration of 1 h.

Following the filtration test, the membrane was cleaned through backwashing. After cleaning, the pure liquid’s permeate flux (*J*_v1_) was remeasured. The reversible flux recovery rate after the first cycle (*F*_*R*1_) was calculated using Equation (6).

(6) $$F_{R1}=\frac{J_{V1}}{J_{V0}}\times 100\%$$

In the second and third cycles, the permeate fluxes of the pure liquid at the end of cleaning were recorded as *J*_v2_ and *J*_v3_, respectively, and the reversible flux recovery rates were *F*_*R*2_ and *F*_*R*3_, respectively.

## 3. Results and Discussion

### 3.1. Physical–Chemical Characterization of the HNTs

**Figure 1:**
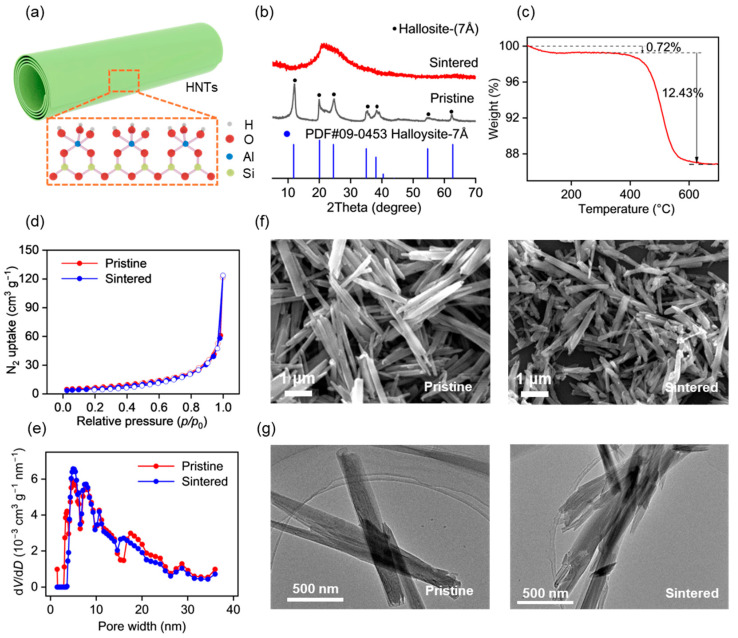
Characterization of HNTs before and after sintering. (**a**) An illustration of the hollow tubular morphology and chemical structure; (**b**) PXRD patterns; (**c**) TG curves; (**d**) nitrogen adsorption–desorption isotherms and (**e**) the relevant pore size distribution; and (**f**) SEM and (**g**) TEM images.

To investigate the crystalline transformation and macro/microstructural changes in the HNTs before and after sintering at 600 °C during the ceramic membrane manufacturing process, we analyzed them using PXRD, BET, SEM, and TEM. Typically, HNTs possess a hollow tubular structure, composed of alternating layers of alumina and silica [41,42,43,44,45]. These layers bent and rolled due to hydration, forming multilayered tubes (Figure 1a). After calcination at 600 °C, the HNTs underwent a transformation into an amorphous structure (Figure 1b), primarily due to the dihydroxylation of the HNTs into meta-Al_2_Si_2_O_5_ between 400 °C and 600 °C (Figure 1c). This transformation was able to improve the interfacial compatibility between the HNTs and the support, as well as γ-Al_2_O_3_, thereby preventing the detachment of the separation layer.

Although the crystal structure of the HNTs changed before and after calcination, their nitrogen adsorption and desorption behaviors did not significantly change. The isotherms remained of type III (Figure 1d), with specific surface areas of 24 m^2^/g and 19 m^2^/g, respectively, and similar pore size distributions (Figure 1e). The morphological changes in the HNTs after thermal calcination at 600 °C were examined using scanning electron microscopy (SEM) and transmission electron microscopy (TEM). The hollow tubular structure of the HNTs remained unchanged before and after sintering, indicating that their physical structure was stable after calcination at 600 °C, while maintaining a high porosity (Figure 1f,g).

### 3.2. The Morphology and Characterization of the Properties of the High-Flux γ-Al_2_O_3_ UF Membranes

The HNTs were utilized to prepare a high-flux intermediate layer, and the spray-coating time was a crucial factor affecting the thickness and integrity of the intermediate layer. The HNTs suspension was sprayed onto the support for 10 s, 30 s, 50 s, 70 s, and 90 s, respectively, and then sintered at 600 °C. As shown in Figure S2, the appearance of the HNTs-derived intermediate layers under spray-coating times of 10 s, 30 s, and 50 s was uniform and continuous, while those coated for 70 s and 90 s exhibited a number of visible irregularly shaped spots, which may have been caused by uneven detachment due to the excessively thick coating. Therefore, in this study, the HNTs intermediate layers were prepared using a spraying time of less than 50 s.

**Figure 2:**
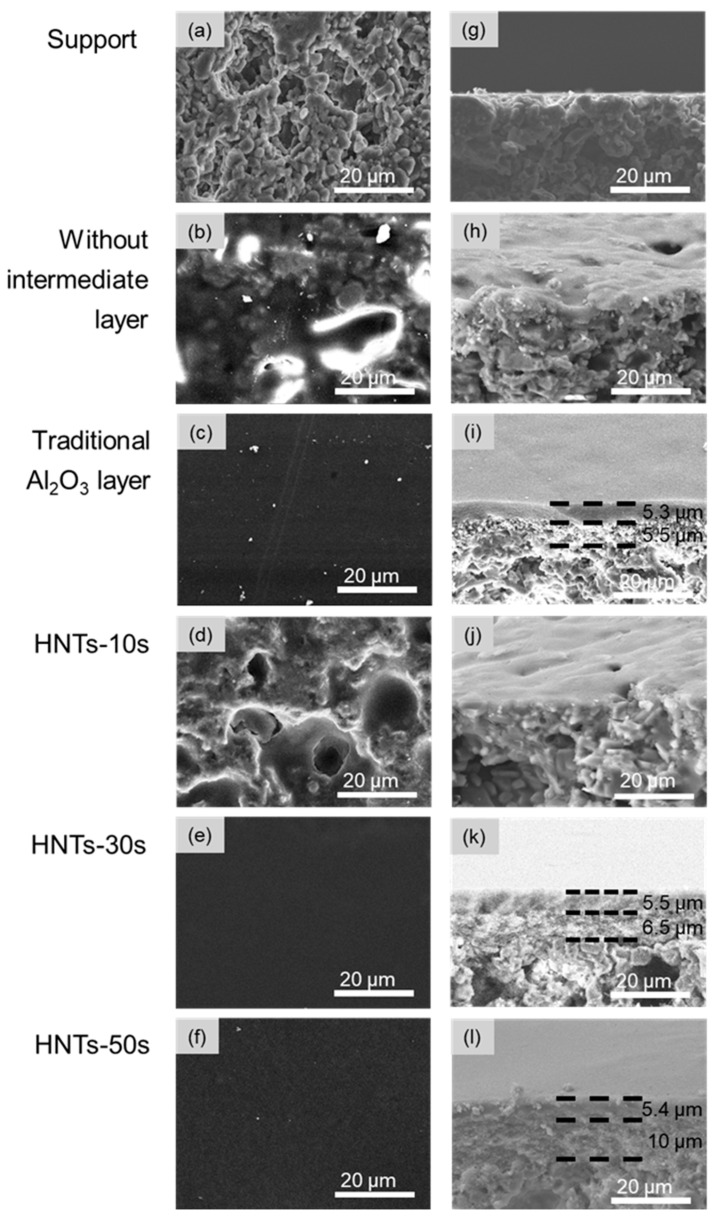
Surface (**a**–**f**) and cross-sectional (**g**–**l**) SEM images of γ-Al_2_O_3_ UF membranes developed on different intermediate layers. (**a**,**g**) show the support as a control; (**b**,**h**) show the γ-Al_2_O_3_ UF membranes developed directly on the support and (**c**,**i**) show the γ-Al_2_O_3_ UF membranes developed on the conventional α-Al_2_O_3_ intermediate layers as a control; and (**d**,**j**), (**h**,**k**), and (**f**,**l**) show the γ-Al_2_O_3_ UF membranes developed on the HNTs-mediated intermediate layers with varying spraying coating durations of 10 s, 30 s, and 50 s, respectively.

Surface and cross-sectional SEM imaging was employed to investigate the microstructural influence of the HNTs-derived intermediate layer on the γ-Al_2_O_3_ separation layer. Surface imaging revealed that the support exhibited a rough and uneven texture, with micron-sized macropores resulting from particle stacking (Figure 2a). When the γ-Al_2_O_3_ separation layer was directly sprayed onto the support without an intermediate layer, significant macroporous defects remained on the membrane’s surface, and severe penetration of γ-Al_2_O_3_ into the support layer was observed (Figure 2b,h), which led to the failure of the γ-Al_2_O_3_ separation layer to be efficiently constructed. Therefore, the construction of a high-quality intermediate layer is generally critical to the formation of ceramic ultrafiltration and nanofiltration separation layers. Precisely for this reason, the construction of the conventional α-Al_2_O_3_ intermediate layers could effectively ensure the formation of high-quality γ-Al_2_O_3_ separation layers, despite the cumbersome fabrication process (Figure 2c,i), in which the layer boundary was clearly visible. However, as a next-generation strategy for intermediate-layer construction, the use of HNTs not only can reduce the complexity of the conventional approaches but also can mitigate the flow resistance induced by the intermediate layer. The surface (Figure 2d–f) and cross-sectional (Figure 2d–f) images of the HNTs-mediated γ-Al_2_O_3_ UF membranes revealed that the spraying duration for the HNTs suspension was critical to the formation of the HNTs-intermediate layers, as well as the formation of the γ-Al_2_O_3_ UF layer developed on them. The γ-Al_2_O_3_ membranes derived from the HNTs-10s intermediate layer still exhibited macroporous defects (Figure 2d,j), suggesting that a spraying duration of 10 s was insufficient to fully cover the macropores of the support. In contrast, the surface of the γ-Al_2_O_3_ membranes derived using the HNTs-30s and HNTs-50s intermediate layers were uniform and continuous, with no discernible defects (Figure 2e,f). Meanwhile, the layer boundaries were clearly visible, and the thickness increased with an increase in the spraying duration (Figure 2k,l). Moreover, after the formation of high-quality HNTs intermediate layers of an excessive thickness, γ-Al_2_O_3_ UF layers can be reliably fabricated via the dip-coating method, achieving a uniform thickness and high reproducibility. It is worth noting that a spraying duration of 30 s for the HNTs suspension was already sufficient for the formation of an intermediate layer given that the transport resistance would increase with the thickness of the intermediate layer.

**Figure 3:**
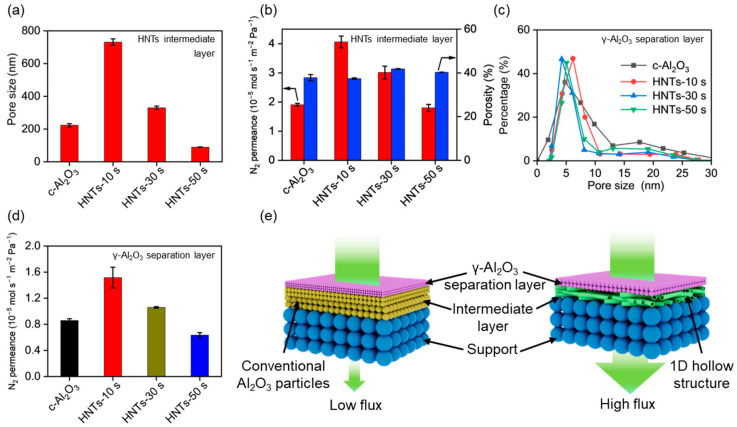
Performance characterization of HNTs-derived intermediate layers and the corresponding γ-Al_2_O_3_ separation layers constructed on top of them. The (**a**) average pore size and (**b**) N_2_ permeance and porosity of the HNTs-derived intermediate layers with different spraying times. (**c**) The pore size distribution, (**d**) the nitrogen permeance, and (**e**) an illustration of the structure of the high-flux HNTs-mediated γ-Al_2_O_3_ UF membranes and a comparison with the conventional case.

To study the effect of the HNTs-derived intermediate layer on the mass transport performance of the γ-Al_2_O_3_ separation layer further and optimize the spray-coating duration further, the pore size and gas permeation properties were probed for the intermediate layer and the γ-Al_2_O_3_ separation layer. The average pore size of the as-prepared intermediate layers before the deposition of the γ-Al_2_O_3_ separation layer is shown in Figure 3a. The conventional intermediate layer developed using multiple α-Al_2_O_3_ layers (c-Al_2_O_3_) showed an average pore size of 200 nm, which made it suitable for the deposition of the γ-Al_2_O_3_ separation layer. Meanwhile, the average pore size of the HNTs-derived intermediate layers decreased sharply with an increase in the spray-coating duration, from 731 nm for HNTs-10s to 89 nm for HNTs-50s, suggesting rapid and efficient construction of the transition layer. Therefore, HNTs-30s appeared to be the optimal spraying duration for the rapid construction of the transition layer. As anticipated, the HNTs-derived intermediate layers exhibited a higher nitrogen permeability and porosity (Figure 3b). The porosity of the support, c-Al_2_O_3_, and HNTs-30s was 37.8%, 37.4%, and 41.8%, respectively. Due to the hollow tubular structure of the HNTs, the presence of the HNTs interlayer increased the overall porosity of the membrane by nearly 4%, which may have significantly reduced the mass transfer resistance across the membrane. This finding demonstrates that the incorporation of the HNTs transition layer not only avoided compromising the overall membrane’s porosity but also significantly enhanced it—a phenomenon rarely observed in the conventional transition layer architectures. Typically, the porosity decreases as the pore size of the transition layer diminishes, making this counterintuitive enhancement a notable deviation from the established trends in membrane design.

After the deposition of the γ-Al_2_O_3_ separation layer onto the different intermediate layers, the pore size distribution was analyzed via a nanopermporometry test. The average pore size and pore size distribution of the γ-Al_2_O_3_ membranes deposited onto the intermediate layers of c-Al_2_O_3_, HNTs-30s, and HNTs-50s were almost the same, with an average pore size value of 5.1 nm independent of the intermediate layers (Figure 3c), which is consistent with the values reported for classical γ-Al_2_O_3_ membranes [46]. However, the γ-Al_2_O_3_ membranes deposited onto the HNTs-10s intermediate layer exhibited a marginally increased pore diameter of 6.2 nm, alongside discernible macroporous defects, possibly due to the nonhomogeneity and low quality of the HNTs-10s-derived intermediate layer, as confirmed hereinbefore (Figure 2d,j and Figure 3a,b). A comparison of the N_2_ permeance, as shown in Figure 3d, revealed that the γ-Al_2_O_3_ membranes deposited onto HNTs-derived intermediate layers with the optimal spraying duration (HNTs-30s) demonstrated a higher N_2_ permeance of 1.06 × 10^−5^ mol s^−1^ m^−2^ Pa^−1^ in comparison to that in those deposited on c-Al_2_O_3_ (0.86 × 10^−5^ mol s^−1^ m^−2^ Pa^−1^). This phenomenon once again validates the advantages of constructing γ-Al_2_O_3_ separation layers based on HNTs-derived transition layers, which not only shortens the construction process for the transition layers but also reduces the overall transport resistance in the membrane structure, as illustrated in Figure 3e.

### 3.3. The Liquid Permeability of the HNTs-Mediated γ-Al_2_O_3_ UF Membranes

**Figure 4:**
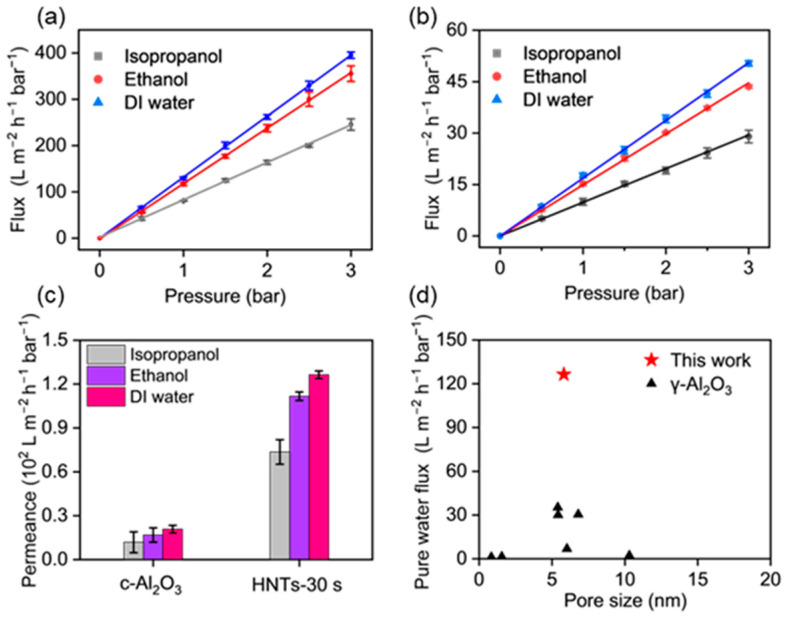
The variation in the liquid permeate flux with pressure for the γ-Al_2_O_3_ UF membranes developed on intermediate layers of (**a**) HNTs-30s and (**b**) c-Al_2_O_3_. (**c**) A comparison of their liquid permeability. (**d**) A comparison of the pure water permeability of HNTs-mediated γ-Al_2_O_3_ UF membranes with reported values.

The permeability of the as-prepared γ-Al_2_O_3_ UF membranes with the optimized HNTs-derived intermediate layer (HNTs-30s) for both an organic solvent and pure water was tested using a home-made cross-flow device to evaluate the possible application of such membranes in CMP waste liquids. As shown in Figure 4a,b, the liquid permeation fluxes of the γ-Al_2_O_3_ UF membranes developed on HNTs-30s were higher than those for c-Al_2_O_3_ under the same conditions. The permeation fluxes for pure isopropanol, ethanol, and water with HNTs-30s were 73, 111, and 126 L h^−1^ m^−2^ bar^−1^, respectively, whereas the corresponding fluxes for c-Al_2_O_3_ were 24, 36, and 51 L h^−1^ m^−2^ bar^−1^, respectively. Both membranes’ fluxes followed a pattern of water > ethanol > isopropanol, which could be attributed to the different viscosities of these liquids (Figure 4c). In addition, a comparison with the values reported for γ-Al_2_O_3_ UF membranes revealed that the HNTs-mediated γ-Al_2_O_3_ membranes in this study exhibited outstanding pure water permeability with a similar range of pore sizes (Figure 4d) [47,48,49,50], once again validating the advantages of the HNTs-mediated γ-Al_2_O_3_ UF membranes.

### 3.4. CMP Waste Liquid Treatment Using the γ-Al_2_O_3_ UF Membranes

**Figure 5:**
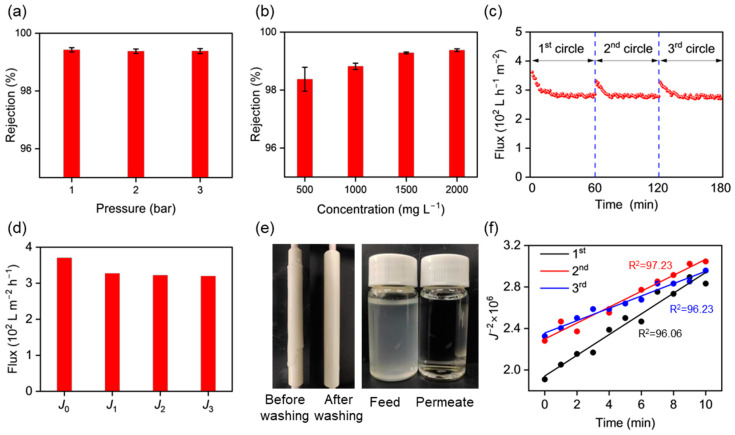
The filtration performance of γ-Al_2_O_3_ UF membranes in simulated CMP wastewater. The effect of (**a**) operation pressure and (**b**) concentration on the rejection of the SiO_2_ particles. (**c**) Circling filtration tests and (**d**) the reversible flux during each cycle. (**e**) Digital photos of the membranes before and after washing during each cycling filtration test, together with the appearance of the feed and the permeate solution. (**f**) The evolution of the permeate flux as a function of the filtration time for different filtration cycles (1st, 2nd, and 3rd). The data were fitted using the cake layer model based on the Hermia model, with the corresponding correlation coefficients (R^2^).

CMP wastewater is typically characterized by high turbidity and alkalinity, containing a large number of abrasive particles, primarily SiO_2_ particles, with an average diameter of about 100 nm. If these components can be recovered, this would not only reduce environmental pollution but also lower the cost of the CMP process. To evaluate the potential of high-flux γ-Al_2_O_3_ UF membranes for CMP wastewater treatment, dispersions of SiO_2_ particles with sizes of 50–100 nm in water were used to simulate CMP wastewater and tested at varying pressures (0–3 bar) and concentrations (0–2000 mg L^−1^). At a constant feed concentration of 2000 mg L^−1^, no significant effect on the retention rate was observed with an increase in the transmembrane pressure, with the retention rates remaining around 99.4% (Figure 5a). This phenomenon suggests that the as-prepared high-flux γ-Al_2_O_3_ UF membranes were suitable for the treatment of the simulated CMP wastewater due to the high degree of matching between the pore size and the particle size, ensuring minimal penetration or clogging. Meanwhile, the feed concentration was found to slightly affect the retention rate in the range of 500–2000 mg L^−1^, where the retention rate was increased from 98.4% to 99.4% (Figure 5b). In addition, to verify the reusability of this type of membrane, cycling tests were conducted at an operating pressure of 3 bar, as shown in Figure 5c. It was observed that during the first filtration cycle, the flux decreased sharply from an initial value of 361 L h^−1^ m^−2^ and leveled off after 1 h, with a significantly high level of 280 L h^−1^ m^−2^. This decrease in flux was attributed to the rise in the permeate resistance caused by the particulate matter being trapped by the membrane and deposited onto its surface and in the pore channels. Nevertheless, after backwashing, the flux could be recovered to a high level of >85%, indicating that the membrane had a good cycling stability (Figure 5c,d). In every filtration cycle, the flux can be recovered, and the membrane’s surface can be fully cleaned using a high-quality, clear, and transparent permeate (turbidity, ~0 NTU) that can be reused (Figure 5e). Mechanistic studies on the fouling behavior during the filtration process were conducted using the Hermia model (Figure 5f). Based on the principles of pore blocking, Hermia et al. proposed four different blocking models for explaining filtration clogging mechanisms: a complete pore blocking model (M1), an internal pore blocking model (M2), an intermediate pore blocking model (M3), and a cake layer model (M4). During the three-cycle filtration processes, the fouling behavior exhibited the highest fitting accuracy with model M4 (Figure S3). This was attributed to the fact that during the filtration of the SiO_2_ dispersion, particulate matter mainly accumulated on the membrane’s surface, forming a cake layer. However, due to the presence of irreversible fouling, other fouling behaviors were still observed during the first filtration cycle.

**Figure 6:**
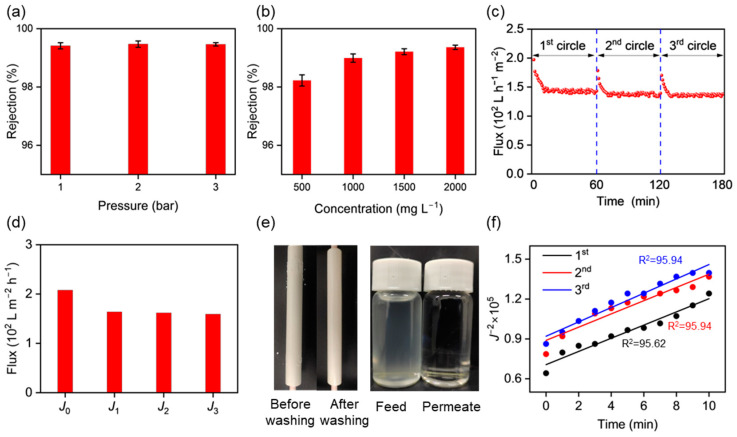
The filtration performance of γ-Al_2_O_3_ UF membranes on simulated isopropanol CMP waste. The effect of (**a**) operation pressure and (**b**) concentration on the rejection of the SiO_2_ particles. (**c**) Cycling filtration tests and (**d**) the reversible flux during each cycle. (**e**) Digital photos of the membranes before and after washing during each cycling filtration test, together with the appearance of the feed and the permeate solution. (**f**) The evolution of the permeate flux as a function of the filtration time for different filtration cycles (1st, 2nd, and 3rd). The data were fitted using the cake layer model based on the Hermia model, with the corresponding correlation coefficients (R^2^).

To investigate the possible applications of such high-flux γ-Al_2_O_3_ UF membranes under harsher conditions, such as an organic solvent system in the semiconductor industry, filtration tests for SiO_2_ particulate matter in isopropanol and ethanol were also conducted (Figure 6 and Figure S4). Similar operation conditions were adopted, and the γ-Al_2_O_3_ UF membranes also demonstrated superior separation performance, cycling stability, and antifouling properties due to the design of the high-flux, robust structure. The retention of SiO_2_ in isopropanol at a concentration of 2000 mg L^−1^ reached 99.4% under a constant pressure of 3 bar. The reversible flux recovery of the membrane after three cycles of filtration was maintained at >80%. The fouling behavior also exhibited the highest fitting accuracy with model M4 (the filter cake layer model), similar to the filtration process in the water and ethanol systems. This once again validated the universal anti-fouling strategy of periodic backwashing within the membrane. Therefore, the high-flux γ-Al_2_O_3_ UF membranes constructed in this work exhibited solvent-agnostic separation and fouling control, positioning them as a universal, scalable solution for multi-component CMP waste remediation.

## 4. Conclusions

In this study, we successfully developed a novel strategy for constructing HNTs-mediated high-flux γ-Al_2_O_3_ UF membranes for semiconductor wastewater treatment. These γ-Al_2_O_3_ UF membranes with optimized HNTs-derived intermediate layers exhibited an outstanding separation performance for CMP wastewater treatment, as well as organic solvent systems. In the filtration tests on the CMP waste liquid, a permeate flux of approximately 50–100 L h^−1^ m^−2^ bar^−1^ and a retention rate exceeding 99.3% were found. The cycling performance tests and the study of the fouling mechanisms demonstrated that the membrane exhibited good stability and a good anti-fouling performance in the different liquid systems. These findings highlight the potential application of the prepared high-flux γ-Al_2_O_3_ UF membranes in CMP wastewater treatment. This work not only developed a scalable and energy-efficient fabrication protocol for ceramic UF membranes for semiconductor wastewater treatment, offering more efficient and cost-effective wastewater treatment solutions, but also established fundamental guidelines for the design of nanostructured transition layers in advanced separation membranes.

## Figures and Tables

**Table 1:** Detailed information on the γ-Al_2_O_3_ ceramic membranes prepared.

Membrane Code	Intermediate Layer	Spray-Coating Time (s)
Support	-	-
c-Al_2_O_3_	α-Al_2_O_3_	-
HNTs-10s	HNTs	10
HNTs-30s	HNTs	30
HNTs-50s	HNTs	50
HNTs-70s	HNTs	70
HNTs-90s	HNTs	90

## Data Availability

The data that support the findings of this study are available from the corresponding author upon reasonable request.

## Supplementary Materials

The following supporting information can be downloaded at: https://www.mdpi.com/article/10.3390/membranes15050130/s1, Figure S1: Bubble-pressure method for testing Support (a) nitrogen flux-pressure curve and (b) pore size distribution; Figure S2: Appearance of HNTs interlayer after sintering for spraying times of 10s, 30s, 50s, 70s and 90s; Figure S3: Fouling Mechanisms study of HNTs-30s for three filtration processes of simulated CMP wastewater: (a) M1, (b) M2, (c) M3, and (d) M4 flux functions with time; Figure S4: HNTs-30s filtration tests on simulated CMP ethanol wastewater: (a) cycle stability test, (b) pure water flux test, (c) filtration effect electronic photo and membrane cleaning before and after washing, the effect of (d) pressure and (e) feed concentration on retention rate, (f) pollution mechanism study for three filtration processes of simulated CMP ethanol wastewater flux functions with time; Figure S5: Study of the contamination mechanism of S-H30-M during three filtrations of simulated CMP ethanol wastewater: (a) M1, (b) M2, (c) M3 and (d) M4 flux functions as a function of time; Figure S6: Fouling Mechanisms study of HNTs-30s for three filtration processes of simulated CMP isopropanol wastewater: (a) M1, (b) M2, (c) M3 and (d) M4 flux functions with time; Table S1: Comparison of the performance of γ-Al2O3 membranes with other literature; Table S2: Correlation coefficients R2 (%) for HNTs-30s after different model fits in the simulation of three filtrations of CMP wastewater; Table S3: Correlation coefficients R2 (%) of HNTs-30s after different model fits in simulating three filtrations of CMP ethanol wastewater; Table S4: Correlation coefficients R2 (%) for HNTs-30s after different model fits in the simulation of three filtrations of CMP isopropanol wastewater.
