# Supplementary material for: Halloysite-Nanotube-Mediated High-Flux γ-Al2O3 Ultrafiltration Membranes for Semiconductor Wastewater Treatment
Source: Membranes (Basel). 2025 Apr 27;15(5):130. doi: 10.3390/membranes15050130 (PMC12112845; doi:10.3390/membranes15050130)
Supplement: Supplementary file 1 [file membranes-15-00130-s001.zip › membranes-3572918-supplementary.pdf]

Supplementary Information

Halloysite-Nanotube-Mediated High-Flux γ-Al_2O_3 Ultrafiltration Membranes for Semiconductor Wastewater Treatment

Shining Geng 1, Dazhi Chen 1, Zhenghua Guo 1, Qian Li 1, Manyu Wen 1, Jiahui Wang 1, Kaidi Guo 1,2, Jing Wang 2, Yu Wang 3, Liang Yu 1,2,4,*, Xinglong Li 5 and Xiaohu Li 6,*

[1] *Beijing Key Laboratory of Photoelectronic/Electrophotonic Conversion Materials, Key Laboratory of Cluster Science, Ministry of Education, Advanced Technology Research Institute (Jinan), Beijing Institute of Technology Chongqing Innovation Center, Advanced Research Institute of Multidisciplinary Science, School of Chemistry and Chemical Engineering, Beijing Institute of Technology, Beijing 100081, China*

[2] *Beijing Institute of Technology, Zhengzhou Academy of Intelligent Technology, Zhengzhou 450000, China*

[3] *Chongqing Advanced Materials Institute (CAMI), Chongqing 408000, China*

[4] *Beijing Institute of Technology, Zhuhai 519088, China*

[5] *Guangdong Guoyu Equipment Co., Ltd., Foshan 528222, China*

[6] *School of Materials Science & Engineering, Beihang University, Beijing 102206, China*

*__Corresponding author__

E-mail address: liangyu@bit.edu.cn (L.Y.); xiaohuli@buaa.edu.cn (X.L.)

Contents

Contents	page number
ESI-1 Basic characterization of Al_2O_3 support	S3
ESI-2 Appearance of membranes	S4
ESI-3 Comparison of the performance with other literature	S4
ESI-4 Study of fouling mechanisms in simulated CMP wastewater	S5
ESI-5 Filtration performance of particles in simulated ethanol wastewater	S5-S8
ESI-6 Study of fouling mechanisms in simulated CMP isopropanol wastewater	S8-S9
References	S9

ESI-1 Basic characterization of Al_2O_3 support

To investigate the effect of different supports on the final membrane properties and structure, the pore size distribution and nitrogen flux of the support were first characterized. As shown in Fig. S1, the support exhibited a broad pore size distribution ranging from 400 to 1800 nm, with a nitrogen permeability of 4.2×10^{-5} mol s^{-1} m^{-2} Pa^{-1}. The high nitrogen permeability indicated relatively low mass transfer resistance, suggesting that after the deposition of the top layer, the support contributed minimally to the overall resistance of the membrane. However, due to its large pore size, sol particles could easily infiltrate the support's interior during the sol-gel process[18, 19], making it challenging to form a continuous and defect-free separation layer on the surface. Therefore, an intermediate layer was necessary to bridge the separation layer and the support during membrane fabrication.

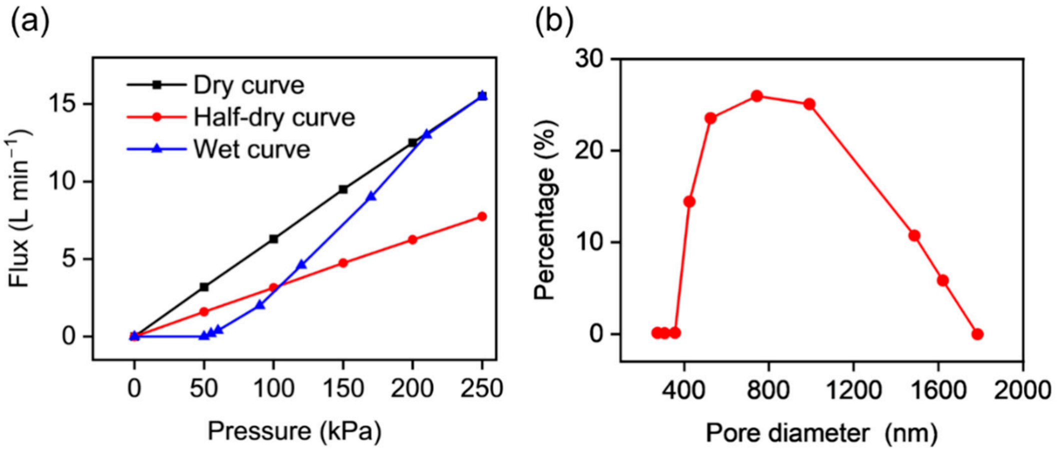

Figure S1. Bubble-pressure method for testing Support (a) nitrogen flux-pressure curve and (b) pore size distribution

ESI-2 Appearance of membranes

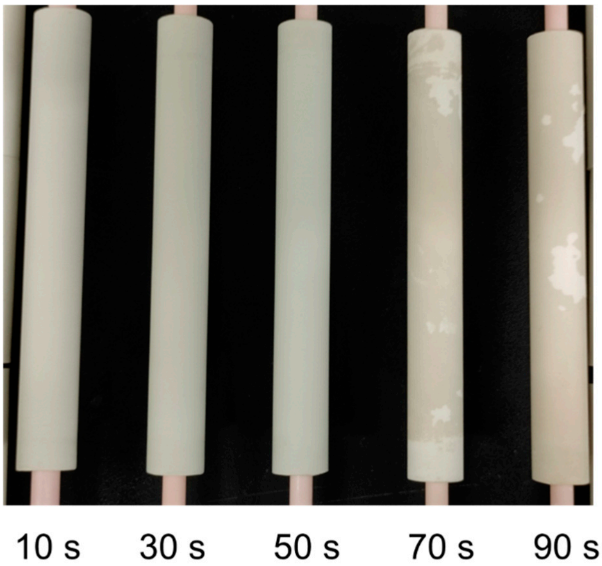

Figure S2. Appearance of HNTs interlayer after sintering for spraying times of 10s, 30s, 50s, 70s and 90s

ESI-3 Comparison of the performance with other literature.

Table S1. Comparison of the performance of γ-Al_2O_3 membranes with other literature.

Membrane code	Pore size (nm)	Porosity (%)	Pure water flux (L m^{-2} h^{-1} bar^{-1})
γ-Al_2O_3-RMs	5.4	38.4	35.2
γ-Al_2O_3	6.8	41.5	30.4
γ-Al_2O_3	4.6	43.1	68
γ-Al_2O_3-NPs	4.7	37.2	62
γ-Al_2O_3-Bi layer	5	--	72
γ-Al_2O_3-top-0.6μm	7.3	--	96
γ-Al_2O_3-top-0.8μm	6.1	--	83
γ-Al_2O_3-top-1.0μm	4.6	--	68
This work	5.8	41.8	126.4

ESI-4 Study of fouling mechanisms in simulated CMP wastewater

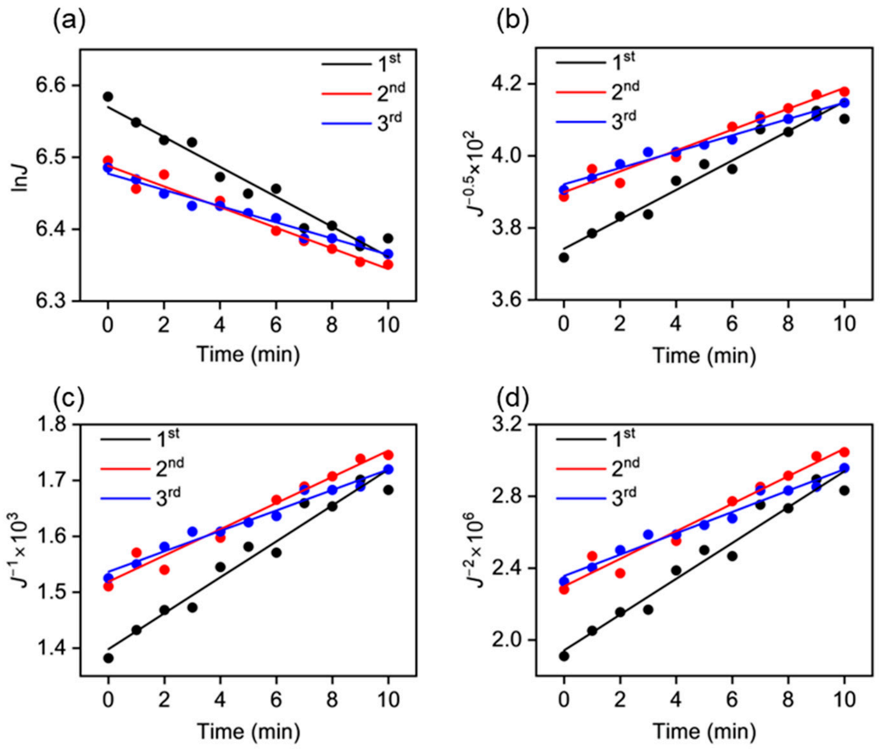

Figure S3. Fouling Mechanisms study of HNTs-30s for three filtration processes of simulated CMP wastewater: (a) M1, (b) M2, (c) M3, and (d) M4 flux functions with time

Table S2. Correlation coefficients R^2 (%) for HNTs-30s after different model fits in the simulation of three filtrations of CMP wastewater

Number of cleaning cycles	M1	M2	M3	M4
1	95.58	95.76	95.89	96.06
2	95.79	95.93	96.05	96.23
3	96.87	96.77	97.08	97.23

ESI-5 Filtration performance of particles in simulated ethanol wastewater

In this work, the retention of SiO_2 particles in ethanol by the HNTs-30s membrane was further investigated. The flux variation over time during ethanol filtration was similar to that observed in water (Fig. S4a). The retention rate of particles in a 2000 mg L^{-1} SiO_2 ethanol dispersion was 99.3% at a constant pressure of 3 bar, and the permeate was clear and transparent with a turbidity of 0 NTU, indicating excellent retention

performance. After the membrane was cleaned and regenerated, no obvious contaminants were observed on its surface (Fig. S4b-c). The membrane was then subjected to cycling tests, and its permeate flux remained stable throughout each cycle, with reversible flux recovery values of 77.9%, 75.5%, and 75.4% for three successive cycles, confirming good stability in filtering SiO_2 from ethanol. The effects of feed concentration and pressure on membrane retention were also investigated (Fig. S4d-e). Consistent with previous findings, transmembrane pressure did not significantly affect retention, while an increase in feed concentration resulted in a higher overall retention rate.

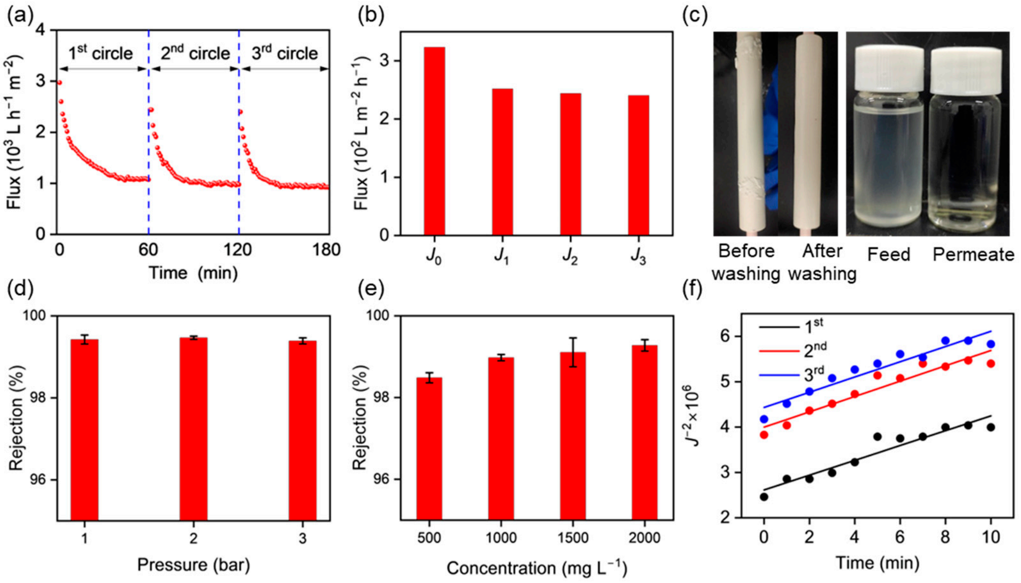

Figure S4. HNTs-30s filtration tests on simulated CMP ethanol wastewater: (a) cycle stability test, (b) pure water flux test, (c) filtration effect electronic photo and membrane cleaning before and after washing, the effect of (d) pressure and (e) feed concentration on retention rate, (f) pollution mechanism study for three filtration processes of simulated CMP ethanol wastewater flux functions with time.

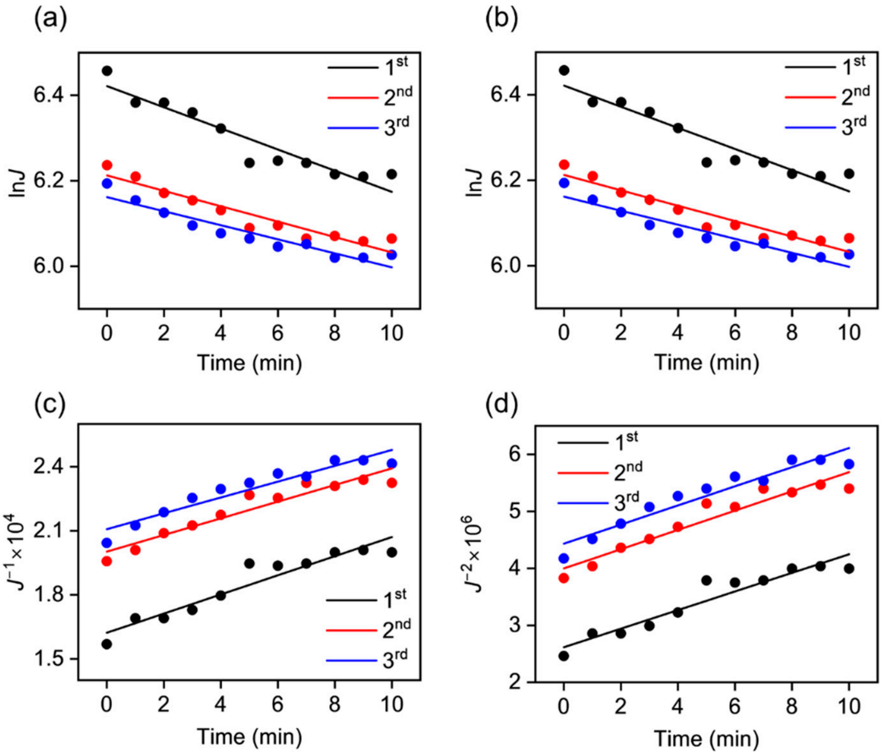

Figure S5. Study of the contamination mechanism of S-H30-M during three filtrations of simulated CMP ethanol wastewater: (a) M1, (b) M2, (c) M3 and (d) M4 flux functions as a function of time

Fouling behavior was further analyzed using the Hermia model (Fig. S4d). Model M4 provided the best fit for all three cyclic filtration processes, which was attributed to the deposition of particulate matter on the membrane surface forming a cake layer during the filtration of SiO_2 dispersion by the S-H30-M membrane. However, due to the presence of irreversible fouling, additional fouling behaviors were observed during the first filtration cycle.

Table S3. Correlation coefficients R^2 (%) of HNTs-30s after different model fits in simulating three filtrations of CMP ethanol wastewater

Number of cleaning cycles	M1	M2	M3	M4
1	89.95	90.36	90.74	91.38
2	90.59	90.97	91.33	91.97
3	90.02	90.56	91.07	92.01

ESI-6 Study of fouling mechanisms in simulated CMP isopropanol wastewater

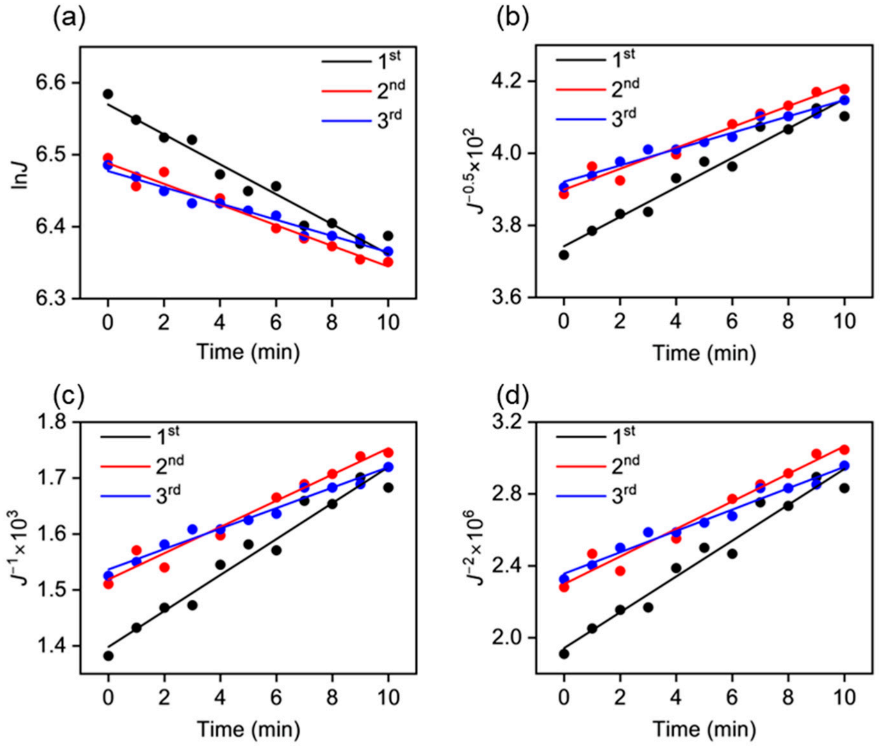

Figure S6. Fouling Mechanisms study of HNTs-30s for three filtration processes of simulated CMP isopropanol wastewater: (a) M1, (b) M2, (c) M3 and (d) M4 flux

functions with time

Table S4. Correlation coefficients R^2 (%) for HNTs-30s after different model fits in the simulation of three filtrations of CMP isopropanol wastewater

Number of cleaning cycles	M1	M2	M3	M4
1	93.18	94.06	94.76	95.62
2	85.87	88.84	90.03	95.94
3	93.30	94.04	94.73	95.94

References

18. Nandi, B.K.; Uppaluri, R.; Purkait, M.K. Preparation and characterization of low cost ceramic membranes for micro-filtration applications. *Appl. Clay Sci.* **2008**, *42*, 102–110. https://doi.org/10.1016/j.clay.2007.12.001.

19. Z.Z. Xuebin Ke, Hongwei Liu, High-flux ceramic membranes with a nanomesh of metal oxide Ke, Z.Z.X.; Liu, H. High-flux ceramic membranes with a nanomesh of metal oxide nanofibers. *J. Phys. Chem. B* **2008**, *112*, 5000–5006.
